# The Impact of Clear Aligner Treatment in Masticatory Function and Temporomandibular Disorders: A Clinical Cohort Pilot Study

**DOI:** 10.3390/healthcare13131541

**Published:** 2025-06-27

**Authors:** Teresa Pinho, Vanessa Marcelino, Maria Gonçalves, Rui M. S. Azevedo, Duarte Rocha, Maria Paço

**Affiliations:** 1 UNIPRO—Oral Pathology and Rehabilitation Research Unit, University Institute of Health Sciences [IUCS], CESPU, 4585-116 Gandra, Portugal; vanessa.k.marcelino@gmail.com (V.M.); duarterocha.95@hotmail.com (D.R.); maria.paco@ipsn.cespu.pt (M.P.); 2 UMIB-Multidisciplinary Biomedical Research Unit, Abel Salazar Institute of Biomedical Sciences (ICBAS), University of Porto, 4050-313 Porto, Portugal; 3 Associate Laboratory i4HB—Institute for Health and Bioeconomy, University Institute of Health Sciences—CESPU, 4585-116 Gandra, Portugal; mprazeres.goncalves@iucs.cespu.pt (M.G.); rui.azevedo@iucs.cespu.pt (R.M.S.A.); 4 UCIBIO—Applied Molecular Biosciences Unit, Translational Toxicology Research Laboratory, University Institute of Health Sciences [1H-TOXRUN, IUCS—CESPU], 4585-116 Gandra, Portugal; 5 UCIBIO—Applied Molecular Biosciences Unit, Forensic Sciences Research Laboratory, University Institute of Health Sciences (1H-TOXRUN, IUCS-CESPU), 4585-116 Gandra, Portugal

**Keywords:** orthodontic stability, orthodontic aligners, masticatory performance, temporomandibular joint dysfunction, jaw function, occlusal dynamics, dental occlusion, chewing efficiency

## Abstract

**Background/Objectives**: This study aimed to explore the functional implications of occlusal changes during clear aligner treatment (CAT) to (a) analyze occlusal changes throughout CAT and the extent of post-treatment occlusal recovery; (b) assess the relationship between post-treatment occlusion and masticatory performance; (c) investigate whether case complexity, facial biotype, and type of malocclusion influence occlusal adaptation and functional outcomes; and (d) evaluate the presence and progression of signs or symptoms of TMDs in patients undergoing CAT. **Methods**: This longitudinal cohort pilot study included 42 individuals who underwent CAT. Occlusion was evaluated at three timepoints: before treatment (T0), at treatment completion (T1), and three months after with night-only aligner use (T2). Masticatory performance was assessed using a two-colored chewing gum test analyzed through colorimetric software. TMD signs/symptoms were assessed using the Diagnostic Criteria for TMD [DC/TMD]. Statistical analysis used non-parametric tests. **Results**: A significant decrease in occlusal contact area was observed during active CAT [*p* = 0.016], which partially recovered at follow-up. Individuals with normal facial proportions (normodivergent) showed more anterior contacts at T1 compared to hyperdivergent individuals [*p* = 0.013]. Masticatory performance remained stable between T1 and T2 [*p* = 0.528]. A weak negative correlation was found between posterior contact number and performance score at T1 [r = −0.378, *p* < 0.05], suggesting that more contacts may be linked to better chewing. No TMD signs or symptoms were detected at any timepoint. **Conclusions**: Although CAT temporarily reduces occlusal contact area, it does not negatively impact chewing efficiency or trigger TMD symptoms. These findings support the functional safety of CAT when treatment is properly planned and monitored.

## 1. Introduction

The advent of digital technology along with the rapid development of biocompatible materials has revolutionized orthodontic treatments, introducing innovative approaches such as clear aligner therapy (CAT) alongside conventional fixed appliances. Clear aligners offer aesthetic and functional benefits, gaining popularity due to their discreet appearance, patient convenience, and reduced treatment-related discomfort compared to conventional fixed appliances. A primary goal of orthodontic treatment is to achieve proper dental alignment and harmonious occlusal relationships, contributing to both functional stability and patient quality of life [1]. Achieving a stable and functional occlusion is critical not only for aesthetic purposes but also for maintaining long-term masticatory function and joint health [1,2,3]. Effective occlusion supports the balanced distribution of occlusal forces, minimizing the risk of temporomandibular disorders (TMDs) and enhancing chewing efficiency, which is crucial for overall masticatory performance [4]. One key occlusal objective is to ensure that contact points are softer anteriorly and stronger posteriorly, promoting stability and functional connectivity within the masticatory system [5]. However, the use of clear aligners presents unique challenges in meeting these objectives due to their full-coverage design, which may induce a “bite-block effect”, limiting natural occlusal settling during treatment [6]. Furthermore, the literature indicates that CAT often fails to establish solid posterior occlusal contacts during active treatment, potentially leading to occlusal imbalances [6]. These issues with posterior contacts may improve during post-treatment settlement; however, there is a lack of consensus on how effectively these contacts stabilize over time [7,8,9,10].

Recent studies have indicated that CAT can lead to alterations in occlusal contacts, potentially impacting masticatory efficiency and occlusal stability [4]. Efficient mastication is crucial for overall oral and digestive health, relying on proper occlusal contacts and coordinated muscle activity. Alterations in occlusion during orthodontic treatment can influence muscle function and masticatory efficiency. Furthermore, there is an ongoing debate regarding the association between orthodontic interventions and the development or exacerbation of TMDs. Some evidence suggests that changes in occlusion may affect temporomandibular joint function, although findings are inconclusive [4]. Factors such as skeletal divergence, individual masticatory force, and occlusal configuration can influence the impact of CAT on TMD, adding complexity to the relationship [11].

So, this study aims to bridge the existing knowledge gap by evaluating not only the occlusal changes during CAT but also their effects on masticatory efficiency and the presence of TMD signs and symptoms. By considering factors such as case complexity, facial biotype, and type of malocclusion, this research seeks to provide valuable clinical insights for healthcare professionals. Understanding these interactions is essential for optimizing both functional and aesthetic outcomes of CAT, ensuring a comprehensive, evidence-based approach to patient care. 

Considering this, and given the scarcity of longitudinal data and persistent ambiguities in the literature, this study aims to explore the functional implications of occlusal changes during CAT. Specifically, it seeks to (a) analyze occlusal changes throughout CAT and the extent of post-treatment occlusal recovery; (b) assess the relationship between post-treatment occlusion and masticatory performance; (c) investigate whether case complexity, facial biotype, and type of malocclusion influence occlusal adaptation and functional outcomes; and (d) evaluate the presence and progression of signs or symptoms of TMDs in patients undergoing CAT.

## 2. Materials and Methods

This is a quantitative, comparative, and observational longitudinal cohort study, without an experimental intervention or randomization. Ethical approval was obtained from the ethical committee of the University Institute of Health Sciences (26/CE-IUCS/2020) on 11 May 2020. This study respected the recommendations of the Helsinki Declaration (2000) and the World Health Organization (WHO) regarding experimentation involving human individuals. Informed consent was obtained from all participants before beginning the study.

### 2.1. Sample

A non-probabilistic convenience sample was recruited from individuals with complete permanent dentition (excluding third molars) undergoing CAT. Participants were recruited at Clínica Manuel Neves, Lda in Porto, and at Clínica Médico-Dentária de São João da Madeira, Lda, Portugal. All treatments were performed a dual specialist in Orthodontics and Pediatric Dentistry, recognized as an Invisalign^®^ Diamond Provider (T.P.). The final sample consisted of 42 individuals (28 female, 14 male), assessed at three timepoints: before CAT (T0), after treatment completion (T1), and three months post-treatment using night-time aligners only (T2). All participants followed the same standardized protocol with fixed timepoints (T0: baseline; T1: end of treatment; T2: 3 months of night-only use).

### 2.2. Inclusion Criteria

Individuals who started and completed CAT, completed an assessment of their masticatory performance at the end of treatment and 3 months after the end, used the night aligners without maladjustments between the end of treatment and 3 months after the end of the treatment, and had available and complete cephalometric analysis.

### 2.3. Exclusion Criteria

Individuals who did not have complete dentition or whose occlusal records were incomplete; presenting (before orthodontic treatment) TMD signs or symptoms, cognitive or neurological changes, identified syndromes, history of trauma and/or tumors in the head and neck, or metabolic diseases affecting the joints and/or muscles; who were on anti-inflammatory, analgesic, or psychiatric medication; or who had suffered from dental pain or periodontal problems in the last year.

### 2.4. Data Collection in TMD Diagnosis

TMDs were assessed clinically using the Diagnostic Criteria for Temporomandibular Disorders (DC/TMD) Axis I [12] at T0, T1, and T2. A preliminary calibration study involving 30 participants (not included in the final sample) was conducted to assess intra- and inter-examiner reliability [9]. Three trained evaluators (MV, DR, TP) independently performed the assessments twice, one week apart. Intraclass correlation coefficients (ICC) exceeded 0.90, indicating excellent reliability.

### 2.5. Data Collection for Masticatory Performance

The masticatory function was evaluated through a masticatory performance test with two-colored chewing gum. Participants sat upright and chewed the two gums (Hue-Check Gum^®^, Kfissia, Greece) [13], one pink and one blue, for 20 chewing cycles. The chewing cycles were counted silently by the operator. The gums were initially moistened in water and bonded together with moderate force. The chewing gum was placed in the patient’s mouth, with the blue side facing the tongue. The chewing sequence should be normal, and so no further instructions were given. After 20 chewing cycles, the operator asked the patient to stop chewing, and the gum was removed from the oral cavity, placed in a new and clear plastic bag, and subsequently flattened with the help of a 1 mm thick plate. On both sides, the specimen was scanned (no later than after the collection due to enzymatic degradation) and saved as an image (JPEG format) [14].

The composite images were then evaluated with a specifically developed program that is freely available (ViewGum software, version 1.4, dHAL Software (https://www.dhal.com/), Kfissia, Greece). The hue variation (VOH) was the measure of mixing calculated by the software. The VOH was considered a measure of masticatory performance because it has a logarithmic association with the number of chewing cycles [15]. A high VOH results from poorly mixed colors through deficient chewing, whereas adequate chewing leads to well-mixed colors and a low VOH. The VOH is considered as the measure of mixing, meaning that the closer to 0, the better the blending of colors and thus the masticatory performance. Information was collected at moments T1 and T2 and registered.

### 2.6. Digital Measurements

After analyzing the clinical records, the characteristics of each participant were collected and evaluated, such as gender and age at the beginning of CAT. Cephalometric tracing, overbite, overjet, and facial biotype (FMA) were also measured. The facial biotype classification was assessed through the FMA angle (formed by the Frankfort and mandibular planes). Facial biotypes were classified as hypodivergent with values equal to or below 22 degrees, normodivergent with values between 23 and 27 degrees, and hyperdivergent with values equal to or above 28 degrees. Intra-oral photographs and 3D scanner Itero^®^ PLY images of the occlusal contacts were collected for each participant corresponding to moments T0, T1, and T2. Subsequently, the intra-oral photographs and images obtained through the intra-oral scanner at T0, T1, and T2 were compared using Itero^®^ software, version 5.9.1.20. The PLY file of each participant had the following characteristics selected in the Itero^®^ software: “open Shell”, “arches combined (arches locked in bite relation)”, and “PLY (color)”. Cephalometric measurements, the number of anterior and posterior occlusal contacts (T0, T1, and T2), and occlusal areas were organized in an Excel file. These data were later used for statistical analysis [16].

To obtain the area and the number of occlusal contacts existing at T0, T1, and T2, the files in PLY format were processed in Meshlab^®^ software, version 2020.03. Quantification of the number of occlusal contacts was also confirmed in ClinCheck^®^ Pro 6.0. Any opposing pair of teeth, maxillary and mandibular, with the same tooth number, counted as one occlusal contact, and the counting was performed on the lower arch. The maximum expected number of occlusal contacts in a twenty-eight-tooth dentition was fourteen. The maximum number of posterior occlusal contacts was eight, and the maximum number of anterior occlusal contacts was six. Third molars were excluded for the area and occlusal contact measurements.

### 2.7. Clinical Assessment and Complexity of the Case

Based on the clinical data [clinical photographs, radiographs, and dental casts], the complexity levels of cases receiving CAT were assessed independently by two professionals. In cases of discrepancy, a third researcher was consulted. The evaluation of case complexity comprises a series of clinical conditions that lead to a final classification of simple, moderate, or complex. This classification considers different parameters: type of dentition, need for surgery, the amount of spacing, crowding, rotations, narrow arches, posterior cross-bite, anteroposterior correction, anterior cross-bite, anterior open bite, deep bite, and need for extraction. Each of these parameters has sub-parameters which were also evaluated.

To aid in this classification, an online service, the Invisalign^®^ evaluation tool, part of the Invisalign^®^ Doctor Site. The orthodontic malocclusion traits were classified as simple, moderate, or complex based on the Invisalign^®^ assessment recommendations. According to the Invisalign^®^ criteria, patients only need one complex problem to classify their case as complex.

### 2.8. Orthodontic Intervention

The individuals were instructed to use each clear aligner for as many hours as possible (20–22 h/day), as instructed by the provider, and only to remove them to eat and during oral hygiene practices. The aligners were changed every seven days from the beginning of the treatments, as recommended by Align^®^ protocols. The control consultations were carried out every two months. Additional aligners were required after T1 to work as a retention method and used only at night to improve the occlusal contacts.

For the present investigation, only one aligner system was used to treat all individuals to reduce confounding factors linked to the material’s mechanical properties. It has indeed already been demonstrated that differences in thermoplastic material composition and thickness result in different flexibilities, force activation, and consequent stress–relaxation behavior [17]. The additional aligners applied after completing the treatment were used without mismatch in the upper and lower arches and only at night.

### 2.9. Statistical Analysis

All statistical analyses were conducted using IBM SPSS^®^ version 29.0 for Windows. Descriptive statistics was performed to estimate frequencies, percentages, means, medians, standard deviations, and ranges. The Shapiro–Wilk test was used to assess the normality of the sample. As the data did not follow a normal distribution, non-parametric tests were applied. To compare occlusal area and contact numbers across T0, T1, and T2, Friedman’s test and the Wilcoxon Signed-Rank test were used. The latter was also applied to compare masticatory performance differences between T1 and T2. Masticatory performance differences between T1 and T2 were evaluated using the Wilcoxon Signed-Rank test. Kruskal–Wallis tests followed by Dunn’s post hoc test with Bonferroni correction were used to compare outcomes by facial biotype, case complexity, molar class, and vertical skeletal pattern. Spearman’s correlation coefficient assessed relationships between occlusal area, anterior/posterior contacts, and masticatory performance at T1 and T2. The significance level was set at α < 0.05.

## 3. Results

### 3.1. Sample Characteristics

Participants were aged between 11 and 45 years [mean age: 22.19 ± 10.51], with 28 females [66.7%] and 14 males [33.3%]. None of the participants exhibited signs or symptoms of TMD. Regarding facial biotype, 20 individuals [47.6%] had a normodivergent biotype, 12 [28.6%] were hyperdivergent, and 10 [23.8%] were hypodivergent. Most participants presented a normal overbite [57.1%] and normal overjet [59.5%]. Clinically, 17 individuals [40.5%] had deep bites, 15 [35.7%] had normal bites, and 10 [23.8%] had open bites. As for case complexity, 6 [14.3%] were classified as simple, 27 [64.3%] as moderate, and 9 [21.4%] as complex.

The sample showed a balanced distribution of facial biotypes and bite types, with most cases classified as moderate complexity with no baseline TMD signs.

### 3.2. Changes in Occlusal Contacts and Areas Throughout the Complete CAT

The comparison of changes in occlusal area, posterior contacts, and anterior contacts during the complete clear aligner treatment (CAT) is presented in Table 1. A statistically significant reduction in occlusal area was observed between the initial (T0) and final (T1) assessments [*p* = 0.016]. However, no significant differences were found in posterior or anterior contacts throughout the treatment.

Occlusal area decreased significantly during treatment, while contact points remained stable.

### 3.3. Comparison of Masticatory Performance at the End of CAT and 3 Months After Night Use Only

When comparing median values of masticatory performance at the end of the CAT (T1) and 3 months after (when only using CA at night—T2), there were no statistically significant differences (Table 2). However, there was a slight reduction in the median values of masticatory performance (VOH variable), which indicates better masticatory performance. All participants followed the same standardized protocol with fixed timepoints (T0: baseline; T1: end of treatment; T2: 3 months of night-only use).

### 3.4. Comparison of the Occlusal Area, Occlusal Contacts, and Masticatory Performance Throughout CAT, According to the Complexity of the Case

At T1, the individuals with simple complexity presented the highest occlusal area values compared to the moderate-complexity and complex cases [*p* = 0.027]. A statistically significant difference in the occlusal area between moderate-complexity and simple cases [*p* = 0.023] was also found. Significant differences were found in posterior contacts at T0, with complex cases showing lower median values than moderate and simple cases [*p* = 0.046] (Table 3). Easier cases tended to retain better occlusal area and contact values throughout treatment compared to more complex ones.

### 3.5. Comparison Between Occlusal Area, Occlusal Contacts, and Masticatory Performance Throughout CAT and According to Facial Biotype

Table 4 compares the occlusal area, anterior contacts, posterior contacts, and masticatory performance according to the facial biotype of individuals who underwent CAT at the three assessment times. Regarding anterior contacts, statistically significant differences were found at T1, with normodivergent individuals presenting a significantly higher number of contacts when compared to hyperdivergent individuals [*p* = 0.013].

Facial biotype influenced anterior contact development, with normodivergent individuals showing more favorable occlusion.

### 3.6. Comparison of Occlusal Area, Occlusal Contacts, and Masticatory Performance Throughout CAT, According to Initial Class Molar Relation

In absolute terms Class I molar individuals had significantly higher anterior contacts than Class II. Dunn’s test showed that these differences are established between the Class I and II groups [*p* = 0.004]. These significant differences were only verified between Class II molar and III individuals [*p* = 0.011]. At T2, Class III molar individuals presented significantly higher values regarding anterior contacts than Class II molar individuals. These differences were statistically significant between the Class I and II molar groups [*p* = 0.024] and between the Class II and III molar groups [*p* = 0.011] (Table 5). So, vertical bite type did not significantly influence occlusal or functional outcomes during treatment.

### 3.7. Comparison Between Occlusal Area, Occlusal Contacts, and Masticatory Performance Throughout CAT, According to the Vertical Bite Trend

According to Table 6, no statistically significant differences were verified between the occlusal area, masticatory performance, and anterior and posterior contacts according to the vertical bite trend (deep, normal, and open bite).

### 3.8. Correlation Between Occlusal Area and Occlusal Contacts and Masticatory Performance

Table 7 shows the correlation between the occlusal area and occlusal contacts with masticatory performance. The number of posterior contacts at T1 showed a negative, weak, and statistically significant correlation with masticatory performance at T1 [*r* = −0.378, *p* < 0.05].

### 3.9. Evaluation of the Presence and Progression of Signs or Symptoms of TMDs

At baseline (T0), none of the participants exhibited signs or symptoms of TMDs. This status remained consistent throughout the treatment period (T1) and at the three-month post-treatment follow-up (T2), with no participants developing TMD signs or symptoms at any assessment point.

## 4. Discussion

Our results showed that CAT led to a significant reduction in occlusal area during active treatment, which is common and expected to be resolved with settling. As suggested by some authors [18], this could be explained by the fact that during the retention period, a functional accommodation of occlusion occurs, leading to an increase in the number of contact points. These results align with other findings that demonstrated that aligner coverage and thickness interfere with natural tooth contact and may induce a transient posterior open bite [19,20]. This “bite-block” effect may lead to anatomical changes and difficulties inherent to the obtention of posterior contact [20], and has been proposed to alter neuromuscular coordination and the mechanical advantage of masticatory muscles [5]. Studies showed that the use of clear aligners for intruding posterior teeth allowed anterior mandibular rotation [21,22,23]. Furthermore, reducing the facial height and improving pogonial projection with correct torque, inclination, and maxillary molar distalization will exacerbate Class III malocclusion [22,24,25,26]. However, throughout the orthodontic treatment, the goal is to reposition the lower front teeth to a more posterior position relative to the upper teeth. As the teeth are moved into their new positions, it is common for the patient to experience more anterior occlusal contacts at the end of the treatment. Nonetheless, our findings revealed no significant decline in masticatory performance—highlighting the neuromuscular adaptability of the stomatognathic system. This functional compensation aligns with the evidence provided by some authors [27], who demonstrated that individuals adapt masticatory patterns according to changes in occlusal loading, maintaining efficiency despite altered dental contact patterns [27]. These results are consistent with our previous investigation into occlusal changes induced by CAT [9], and extend its scope to explore functional outcomes, including masticatory performance and the presence of TMD signs and symptoms. The inclusion of masticatory performance testing and the integration of diagnostic criteria for TMDs at multiple timepoints offer a more complete risk profile for patients undergoing aligner-based treatment, further supporting its scientific and practical relevance.

Interestingly, the results revealed no significant association between facial biotype or malocclusion class and masticatory performance, nor any correlation between case complexity and TMD symptoms. These findings are consistent with previous evidence, showing that facial morphology does not significantly affect functional capacity when occlusal balance is maintained [28,29]. Moreover, despite biomechanical challenges associated with difficult movements (e.g., distalization, extrusion), these did not negatively affect function—likely due to the resilience of the masticatory system and the ability of clear aligners to control tooth movement more precisely when properly planned [20].

The absence of TMD signs or symptoms before, during, or after treatment in our study is particularly significant, given the ongoing debate regarding CAT and its potential association with TMJ disorders [30,31,32]. Our findings are further supported by a systematic review conducted by some authors [4], which assessed the impact of CAT on the masticatory musculature and stomatognathic system. The review concluded that while clear aligners significantly affect the muscles of mastication, alterations in muscle activity and discomfort are typically transient, with no significant long-term changes observed during follow-up [4]. This aligns with our observation of the neuromuscular system’s adaptability during CAT. Additionally, a study [33] evaluated the short-term effects of CAT on pain and surface electromyographic activity of masticatory muscles, finding a temporary reduction in masseter muscle activity at rest after one month of treatment, which returned to baseline levels after three months. This temporary alteration parallels our findings regarding the transient nature of occlusal changes and subsequent functional stability.

Despite the insights gained, this study has some limitations that warrant consideration. In relation to the sample size, since all individuals were recruited based on visits to a clinic, there were no a priori power analysis calculations and so the sample should be interpreted within the limitations of a convenience sample. Therefore, these findings should be interpreted as preliminary and hypothesis-generating, rather than definitive. Furthermore, forming subcohorts that undergo alternative treatments to CAT would allow a better comparison of all variables of interest and increase the validity of the study and conclusions. Larger, multi-center studies are warranted to confirm these results and strength external validity. Additionally, the follow-up period was limited to three months post-treatment; longer-term assessments are essential to evaluate the stability of occlusal and functional outcomes over time. The study also relied on specific methodologies for assessing masticatory performance and TMD symptoms; incorporating additional diagnostic tools, such as electromyography or Magnetic Resonance Imaging, could provide a more comprehensive evaluation of neuromuscular adaptations and temporomandibular joint health. Lastly, while efforts were made to control for confounding variables, individual variations in neuromuscular adaptation and occlusal anatomy may have influenced the outcomes.

From a clinical perspective, this study provides reassurance to orthodontists and general dentists regarding the functional safety of CAT. While esthetic outcomes remain a major motivator for patients, maintaining or improving functional performance is essential for long-term oral health. Our results suggest that structural occlusal changes during CAT do not translate into negative functional consequences, provided that a period of occlusal recovery is allowed after active treatment and a systematic evaluation is performed of function as well as signs and symptoms of TMDs.

## 5. Conclusions

This study demonstrates that CAT induces transient reductions in occlusal area during active treatment, which tend to recover after treatment. These findings are preliminary and based on a pilot cohort study. Occlusal modifications do not impair masticatory performance or contribute to TMD development. The findings underscore the neuromuscular adaptability of the masticatory system and suggest that CAT is a functionally safe orthodontic intervention when appropriately managed. These insights provide valuable information for dental professionals, emphasizing the importance of monitoring temporomandibular joint health as well as occlusal changes and allowing for natural settling post-treatment to ensure optimal functional outcomes. 

## Figures and Tables

**Table 1:** Comparison of occlusal area, posterior contacts, and anterior contacts throughout the complete orthodontic treatment with CA.

	n	*Median [IQR]*	χ^2^	*p*
Occlusal Area T0	42	47.37 [28.85–88.50] *	7.87	0.002
Occlusal Area T1	42	25.20 [15.48–43.57] *		
Occlusal Area T2	42	31.07 [21.19–45.90]		
Posterior Contacts T0	42	8.0 [5.0–8.0]	1.54	0.462
Posterior Contacts T1	42	7.0 [5.0–8.0]		
Posterior Contacts T2	42	8.0 [6.0–8.0]		
Anterior Contacts T0	42	4.0 [2.0–6.0]	0.60	0.743
Anterior Contacts T1	42	4.0 [3.0–5.0]		
Anterior Contacts T2	42	4.0 [2.75–6.0]		

Friedman test results. Changes between each moment were analyzed with Wilcoxon Signed-Rank test and are marked with * if significant. Data are summarized as the median and interquartile range (IQR).

**Table 2:** Comparison of masticatory performance at the end of CAT (T1) and 3 months after using them only at night (T2).

	n	*Median [IQR]*	*z*	*p*
Masticatory performance T1	42	0.133 [0.079–0.231]	−0.631	0.528
Masticatory performance T2	42	0.115 [0.055–0.207]		

Note: Data are summarized as the median and interquartile range (IQR); *Z* = statistics of Wilcoxon Signed-Rank test; *p* = significance level.

**Table 3:** Comparison of occlusal area, occlusal contacts, and masticatory performance according to the case’s complexity.

	Complex [n = 9]	Moderate [n = 27]	Simple [n = 6]	*H*	*p*
Occlusal Area T0	32.36 [15.17–47.41]	51.19 [25.04–89.93]	84.13 [21.28–134.21]	3.81	0.149
Occlusal Area T1	15.66 [8.40–31.48]	25.12 [16.02–50.39] *	41.02 [34.48–48.07] *	7.22	0.027
Occlusal Area T2	33.59 [27.63–45.38]	31.96 [20.01–45.20]	30.09 [16.02–57.78]	0.11	0.949
Anterior Contacts T0	2.0 [0.5–5.5]	4.0 [2.0–6.0]	5.0 [2.0–6.0]	2.15	0.341
Anterior Contacts T1	4.0 [2.5–5.0]	3.0 [3.0–6.0]	5.0 [3.5–5.0]	0.81	0.669
Anterior Contacts T2	4.0 [3.0–6.0]	5.0 [3.0–6.0]	2.0 [0.0–6.0]	1.73	0.421
Posterior Contacts T0	5.0 [3.5–7.5]	8.0 [6.0–8.0]	8.0 [7.25–8.0]	6.18	0.046
Posterior Contacts T1	6.0 [4.5–7.0]	7.0 [5.0–8.0]	7.5 [5.75–8.0]	2.35	0.310
Posterior Contacts T2	8.0 [6.0–8.0]	8.0 [6.0–8.0]	7.0 [5.5–8.0]	0.17	0.917
Masticatory performance T1	0.11 [0.53–0.14]	0.14 [0.08–0.23]	0.13 [0.05–0.18]	1.37	0.505
Masticatory performance T2	0.13 [0.08–0.22]	0.10 [0.05–0.20]	0.19 [0.13–0.47]	3.75	0.154

Note: Data are summarized as the median and interquartile range (IQR); *H* = statistics of Kruskal–Wallis Test; *p* = significance level; * significant difference between the moderate and simple groups [*p* = 0.023].

**Table 4:** Comparison of occlusal area, occlusal contacts, and masticatory performance, according to facial biotype.

	Normodivergent [n = 20]	Hyperdivergent [n = 10]	Hypodivergent [n = 12]	*H*	*p*
Occlusal Area T0	52.53 [35.69–80.50]	38.07 [16.36–91.65]	28.67 [14.57–89.57]	1.46	0.481
Occlusal Area T1	34.77 [13.20–34.77]	19.96 [15.47–43.09]	19.93 [18.10–34.47]	1.69	0.431
Occlusal Area T2	34.90 [20.79–48.54]	25.40 [14.18–38.50]	32.77 [24.77–43.30]	1.78	0.413
Anterior Contacts T0	5.0 [2.25–6.0]	2.0 [0.75–6.0]	3.5 [1.0–6.0]	2.32	0.313
Anterior Contacts T1	4.5 [3.0–6.0] *	2.0 [0.0–4.0] *	3.5 [3.0–5.0]	8.16	0.017
Anterior Contacts T2	5.0 [4.0–6.0]	2.5 [0.75–6.0]	4.0 [2.25–5.75]	2.70	0.259
Posterior Contacts T0	8.0 [5.0–8.0]	8.0 [4.75–8.0]	7.5 [5.5–8.0]	0.30	0.862
Posterior Contacts T1	6.5 [5.0–8.0]	6.5 [5.75–8.0]	7.5 [5.25–8.0]	0.41	0.814
Posterior Contacts T2	7.0 [5.25–8.0]	8.0 [6.25–8.0]	8.0 [6.25–8.0]	1.77	0.412
Masticatory Performance T1	0.15 [0.10–0.28]	0.11 [0.09–0.18]	0.08 [0.06–0.13]	5.72	0.057
Masticatory Performance T2	0.12 [0.08–0.21]	0.10 [0.05–0.26]	0.11 [0.05–0.16]	0.45	0.798

Data are summarized as the median and interquartile range (IQR); *H* = statistics of Kruskal–Wallis Test; *p* = significance level; * significant difference between the normodivergent and hyperdivergent groups [*p* = 0.013].

**Table 5:** Comparison of occlusal area, occlusal contacts, and masticatory performance, according to the initial molar class relation.

Class Molar	I [n = 25]	II [n = 10]	III [n = 7]	*H*	*p*
Occlusal Area T0	56.79 [27.45–95.02]	38.62 [19.33–62.55]	39.52 [9.86–51.19]	3.21	0.201
Occlusal Area T1	34.48 [18.23–48.59]	18.55 [15.47–24.16]	20.85 [9.38–35.01]	3.59	0.166
Occlusal Area T2	30.00 [17.97–42.94]	32.77 [19.74–48.42]	37.50 [28.15–58.07]	1.51	0.468
Anterior Contacts T0	6.0 [3.5–6.0] *	2.0 [0.75–2.25] *	2.0 [1.0–5.0]	12.06	0.002
Anterior Contacts T1	4.0 [3.0–5.5]	2.5 [0.0–3.25] **	5.0 [3.0–6.0] **	10.59	0.005
Anterior Contacts T2	5.0 [3.0–6.0] ***	1.5 [0.75–3.25] ***	5.0 [4.0–6.0] ***	10.13	0.006
Posterior Contacts T0	8.0 [5.5–8.0]	7.5 [5.0–8.0]	7.0 [2.0–8.0]	2.98	0.225
Posterior Contacts T1	6.0 [5.0–8.0]	7.0 [5.75–8.0]	6.0 [3.0–8.0]	0.62	0.735
Posterior Contacts T2	7.0 [6.0–8.0]	8.0 [5.5–8.0]	8.0 [7.0–8.0]	1.55	0.460
Masticatory Performance T1	0.14 [0.08–0.31]	0.09 [0.06–0.12]	0.14 [0.08–0.19]	5.67	0.059
Masticatory Performance T2	0.12 [0.07–0.20]	0.10 [0.05–0.27]	0.08 [0.05–0.24]	0.68	0.710

Data are summarized as the median and interquartile range (IQR); *H* = statistics of Kruskal–Wallis Test; *p* = significance level; * significant difference between the I and II groups [*p* = 0.004]; ** significant difference between the II and III groups [*p* = 0.011]; *** significant difference between the I and II groups [*p* = 0.024], and the II and III groups [*p* = 0.011].

**Table 6:** Comparison of occlusal area, occlusal contacts, and masticatory performance, according to the vertical bite trend.

Vertical Bite Trend	Deep [n = 17]	Normal [n = 15]	Open [n = 10]	*H*	*p*
Occlusal Area T0	48.86 [25.91–76.46]	32.30 [24.58–56.79]	80.26 [19.20–103.88]	1.72	0.423
Occlusal Area T1	18.07 [12.33–41.02]	25.40 [19.02–45.21]	34.77 [17.36–62.08]	2.03	0.361
Occlusal Area T2	33.59 [21.51–42.82]	30.18 [23.70–48.72]	28.19 [13.80–42.29]	0.99	0.609
Anterior Contacts T0	4.0 [2.0–6.0]	3.0 [1.0–6.0]	4.5 [1.75–6.0]	0.72	0.696
Anterior Contacts T1	4.0 [3.0–5.5]	4.0 [3.0–5.0]	3.0 [1.0–5.25]	1.23	0.542
Anterior Contacts T2	5.0 [3.0–6.0]	5.0 [2.0–6.0]	3.5 [0.75–5.0]	2.17	0.338
Posterior Contacts T0	8.0 [6.0–8.0]	6.0 [5.0–8.0]	8.0 [7.25–8.0]	3.96	0.138
Posterior Contacts T1	6.0 [5.5–8.0]	7.0 [5.0–8.0]	6.5 [5.0–8.0]	0.23	0.892
Posterior Contacts T2	7.0 [6.0–8.0]	8.0 [7.0–8.0]	8.0 [5.75–8.0]	3.90	0.143
Masticatory performance T1	0.09 [0.06–0.25]	0.14 [0.08–0.23]	0.14 [0.09–0.16]	0.64	0.725
Masticatory performance T2	0.12 [0.10–0.23]	0.11 [0.05–0.25]	0.08 [0.04–0.14]	3.19	0.203

Data are summarized as the median and interquartile range (IQR); *H* = statistics of Kruskal–Wallis Test; *p* = significance level.

**Table 7:** Correlation between occlusal area, occlusal contacts, and masticatory performance at different treatment moments (Spearman’s correlation coefficient n = 42).

	Masticatory Performance T1	Masticatory Performance T2
Occlusal Area T1	−0.229	
Anterior Contacts T1	−0.0.72	
Posterior Contacts T1	−0.378 *	
Occlusal Area T2		0.049
Anterior Contacts T2		−0.200
Posterior Contacts T2		−0.161

* statistical significance set at *p* < 0.05.

## Data Availability

The data presented in this study are available on request from the corresponding author.

## Abbreviations

The following abbreviations are used in this manuscript:

CAT	Clear aligner treatment
TMD	Temporomandibular disorder
